# Measles-mumps-rubella vaccine at 6 months of age, immunology, and childhood morbidity in a high-income setting: study protocol for a randomized controlled trial

**DOI:** 10.1186/s13063-020-04845-7

**Published:** 2020-12-10

**Authors:** Dorthe Maria Vittrup, Anne Cathrine Lund Laursen, Michelle Malon, Jesper Kiehn Soerensen, Jakob Hjort, Soren Buus, Jannet Svensson, Lone Graff Stensballe

**Affiliations:** 1 The Child and Adolescent Department, The University Hospital Herlev, Borgmester Ib Juuls Vej 25D, 4. Sal, 2730 Herlev, Denmark; 2 The Child and Adolescent Clinic, The Juliane Marie Center, The Danish National University Hospital “Rigshospitalet”, Copenhagen, Capital Region of Denmark Denmark; 3 Department of Clinical Medicine, Health, Aarhus University, Aarhus, Denmark; 4 Department of Immunology and Microbiology, University of Copenhagen, Copenhagen, Denmark; 5 Rigshospitalet, The Juliane Marie Center, Blegdamsvej 9, 2100 Copenhagen East, Denmark

**Keywords:** Measles, MMR, Vaccinology, Immunogenicity, Indirect effects of vaccines, Vaccine efficacy, Vaccine safety, Vaccine schedule

## Abstract

**Background:**

Measles is a highly contagious and serious infection. Before the introduction of vaccination, measles caused yearly epidemics putting vulnerable children at risk of brain damage and death. Despite safe and cost-effective vaccines, measles remains a leading cause of death in children globally. Due to insufficient vaccine coverage and low levels of in utero transferred antibodies from vaccinated mothers, outbreaks of measles in Denmark and other high-income countries are observed at increasing frequency.

The current vaccine was introduced in Denmark in 1987 as a one-shot measles-mumps-rubella vaccine at 15 months, a timing chosen to avoid inhibition of the infant’s immune response by maternal antibodies. One generation later, the MMR vaccinated mothers have lower antibody levels compared to the naturally infected, and their infants are already susceptible at 6 months of age or earlier, thus increasing the risk of epidemics.

**Methods:**

The Danish MMR trial is a double-blind randomized clinical trial recruiting between March 2019 and December 2021 with last patient last visit in February 2022. Altogether *N* = 6500 infants aged 6 months will be randomly assigned to intramuscular vaccination with routine MMR (M-M-R VaxPro) or placebo (solvent only). According to the Danish Childhood vaccination program, all infants will receive a routine MMR vaccination at 15 months of age. At randomization, 1 month later, and 1 month after routine MMR vaccination at 15 months of age, a blood sample is drawn from app. 10% (*N* = 600) of the population. Additionally, hair, saliva, and urine are sampled at randomization. The co-primary study outcomes are immunogenicity 1 month after MMR vaccination at 6 months of age assessed as plaque-reduction neutralization test, and incidence of infectious disease hospitalizations from randomization to 12 months of age. Six weeks post randomization, all participants are interviewed regarding adverse events.

**Trial registration:**

The trial is registered in the EU Clinical Trials Registry. EudraCT registration number: 2016-001901-18. Registered on 14 February 2017.

## Background

Measles is a highly contagious disease with severe complications and WHO has the ambition to eradicate measles, as human species is the only known reservoir [1, 2]. The measles-mumps-rubella vaccine (M-M-R VaxPro) is an effective and safe vaccine routinely used as primary immunization in infants between 9 and 15 months of age [2]. The routine child vaccination schedule in Denmark includes MMR-vaccination (standard dose M-M-R VaxPro [3]) at 15 months and 4 years of age [4]. However, measles-vaccinated mothers have lower levels of measles antibodies than mothers who experienced wild-type measles infection [5]. Thus, infants of MMR-vaccinated mothers might not be passively protected against measles for as long as infants of wild-type-infected mothers. We hypothesize that earlier vaccination may be preferable [5–8]. Older studies have suggested that the vaccine response may be inhibited by maternal antibodies if the MMR-vaccine is administered before 12 months of age, but as maternal antibodies are lower when mothers are MMR-vaccinated in childhood, nowadays maternal antibodies are expected to affect the infant’s antibody response for a shorter duration of infancy [6, 7].

Recent studies have suggested positive indirect (also called heterologous or non-specific) effects from vaccination with live attenuated vaccines on child morbidity and mortality in low-income settings [9] and on child morbidity in high-income settings [10, 11]. Therefore, the present trial also tests the potential effect of an early MMR vaccine on hospitalization caused by infection. Thus, the trial has two co-primary outcomes. First primary outcome is to assess the specific immunity in children vaccinated at 6 (5 to 7) months of age measured as level of measles neutralizing antibodies. The WHO has defined criterions for sufficient immunization [12–14]. Second co-primary outcome is to evaluate potential beneficial indirect effect of early MMR vaccination measured as reduction of infectious disease-related hospitalizations between 6 and 12 months of age.

## Methods/design

The Danish MMR trial is a single-center double-blinded clinical trial randomizing 6500 infants at age 6 months to either M-M-R VaxPro-vaccination (standard-titer measles) [15] or placebo vaccination. Among a subpopulation of 600 children, samples of blood, saliva, urine, and hair are collected. The biological mother is asked to give biological samples including blood, saliva, hair, and urine as well.

Inclusion criteria are gestational age of ≥ 32 weeks, birth weight ≥ 1000 g, and parental signed consent.

Exclusion criteria are primary or acquired immunodeficiencies (including medically induced immune suppression), signs of severe illness or major malformations, no Danish speaking parent, history of anaphylactic, anaphylactoid, or other immediate reactions subsequent to egg ingestion, fructose intolerance, thrombocytopenia, and any coagulation disorder. Children who receive blood or plasma transfusions, or administration of human immune serum globulin within the last 3 months, are excluded. All data collected through the study period are kept in the online double-code secured database REDCap, which is only accessible for study staff. Real-time validation checks are used to uphold validity of clinical data.

### Setting

**Fig. 1:**
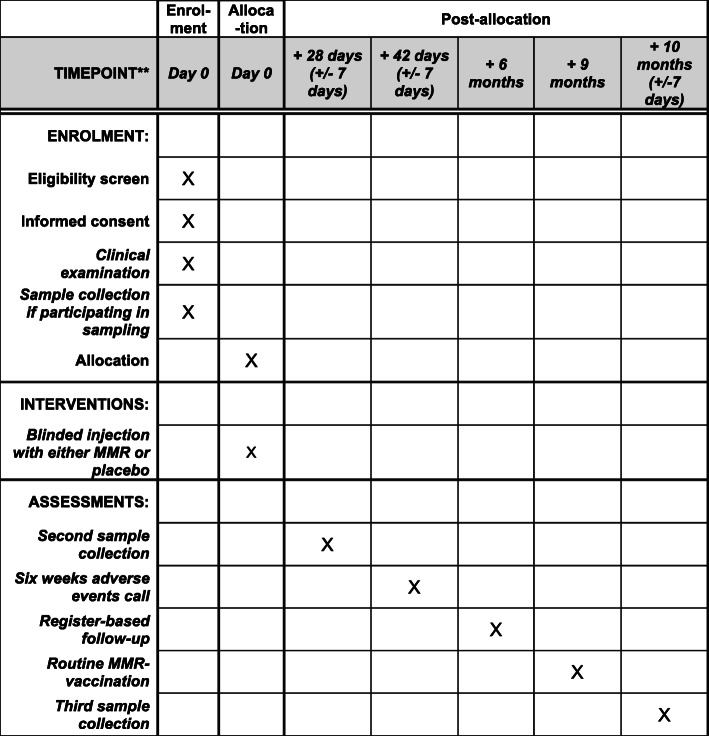
Schematic overview of enrolment, intervention, and assessments. For further details, see Fig. 2

**Fig. 2:**
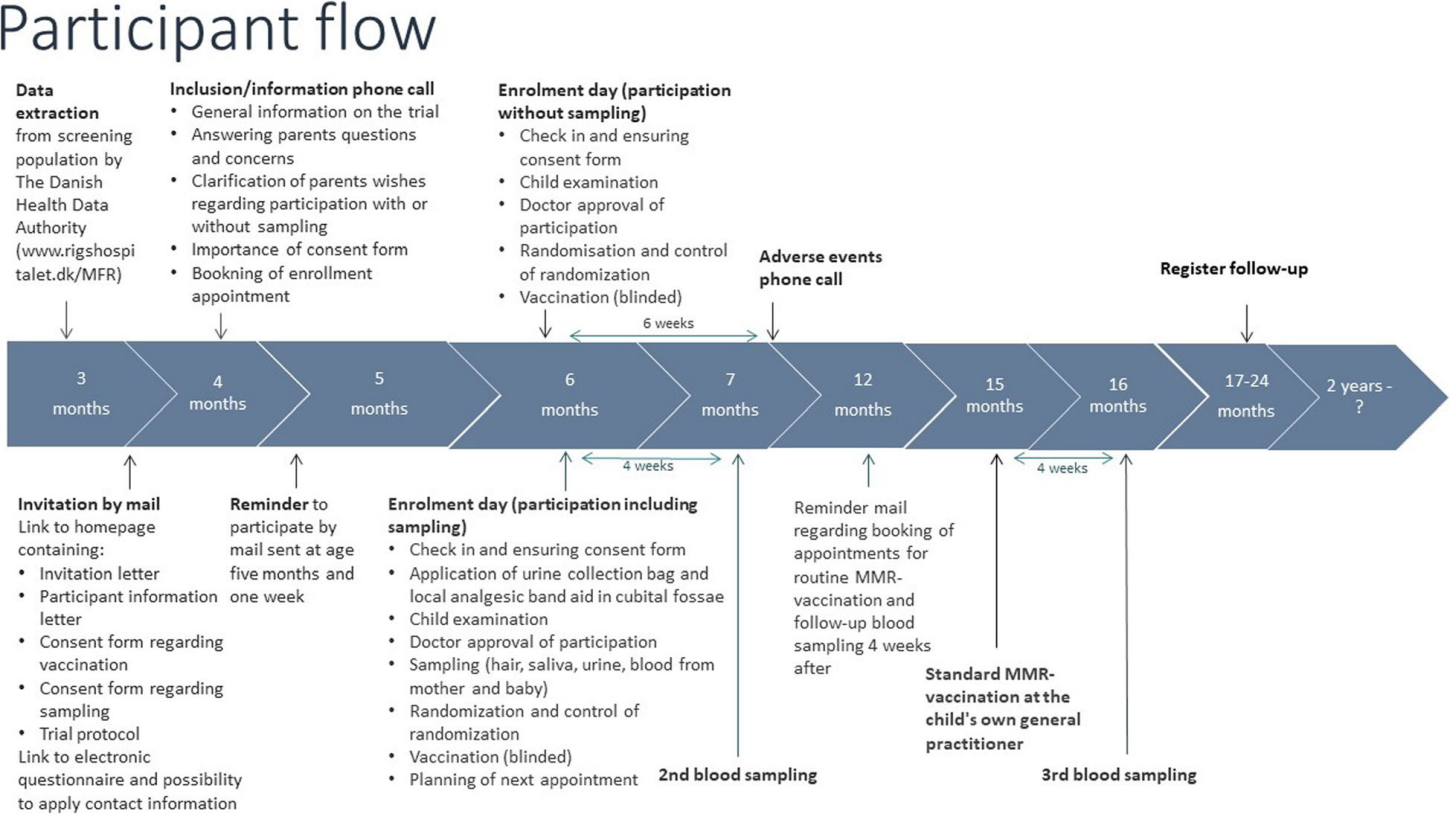
Participant flow in trial. Timeline refers to the age of the child. Each vertical arrow represents a time point, and the accompanying text describes the activities at a certain time point of a participants’ trial participation. Above the timeline are shortly mentioned enrolment day-activities for participation without sampling. Below the timeline, the enrolment day-activities are mentioned for participation including sampling

From February 2019 to December 2021, all mothers of 4-month-old infants born in the Capital Region receive a letter with information about the MMR trial and are invited to participate. The screening population is expected to be > 42,000 infants during the 2-year recruitment period. Invited parents are contacted by phone and offered further information on the phone. If they consent and the child complies with the criteria for participation, a consultation is booked. To ensure participation only by healthy children, the consultation consists of a child examination followed by randomization (Figs. 1 and 2).

At any timepoint during the recruitment period, the study staff aims to optimize public awareness of the study, increase efforts on reaching parents, and improve information in mails and on website [16]. If this strategy leads to fewer participants than powered, optimizing measures will be made timely to avoid extension of recruitment period.

### Interventions

At 6 (5 to 7) months of age participants are randomized to either the intervention group (MMR immunization with M-M-R VaxPro [12]) or to the control group (placebo vaccination with solvent from M-M-R VaxPro) directly in their electronic case report form (e-crf). The manufacturer of M-M-R VaxPro, Sanofi Pasteur MSD [17], manufactures the intervention-injection (M-M-R VaxPro). Thus, package and labeling is the same as used commercially [3]. Participants are randomized to 50% in each group with two-four-six block randomization stratified by sex and prematurity defined as gestational age < 37 weeks.

The random allocation sequence is provided by the e-crf shortly before vaccination and is concealed to all study staff after approval by two study staff members until end of trial defined by last patient last visit (LPLV). Specially trained health professionals assign participants to intervention and vaccinate children. The MMR-group is injected intra-muscularly with 0.5 ml of the M-M-R VaxPro at the anterolateral area of the thigh and the children randomized to the control group are given placebo injection. Both parents and the trial staff are blinded to the intervention as the syringe content is covered with colored tape.

### Outcomes

The trial has two co-primary outcomes

#### Hypotheses

- *First co-primary outcome. Humoral immunity—the evaluation of the M-M-R VaxPro immunogenicity:* The plaque reduction neutralization test (PRNT), which measures the serum dilution capable of preventing 50% of plaque formation induced by measles virus in cell cultures, has been considered the most reliable criterion for the serological evaluation of measles immunity. For PRNT, the protective cut-off dilution is defined to be > 120 [15, 18]. A frequency of 95% seroconversion rate, i.e., children mounting a protective level of humoral immunity according to the abovementioned cutoff value after MMR-vaccination at 6 months of age, will be considered sufficient to implement primary MMR immunization at 6 months in the Danish vaccination program [15, 19].
- *Second co-primary outcome. Potential beneficial indirect effects of MMR at 6 months of age:* Statistically significant 20% decrease in hospitalization due to infection from 6 to 12 months of age in children randomized to receive MMR at 6 months compared to children receiving placebo.

The framework of the trial is equivalence with regard to the first co-primary outcome concerning specific immunity. The WHO has well-defined criteria for sufficient antibody response as a measurement of both IgG, IgM, and plaque reduction neutralization test (PRNT) [15]. Secondary outcomes include immunogenicity of MMR-vaccination measured as IgM and IgG antibodies against measles, mumps, and rubella 1 month following randomization in infants randomized to MMR-vaccine, and 1 month following routine MMR-vaccination at 15 months of age. Maternal antibodies quantified by measles neutralizing antibodies and IgG and IgM to measles, mumps, and rubella prior to randomization will be evaluated. The secondary outcomes also contain other indirect effects of early vaccination including infectious disease hospitalizations within the first 24 months of life and incidence of asthmatic bronchitis and atopic dermatitis. Use of prescribed medication will be assessed regarding anti-asthmatics, topical treatment against atopic dermatitis, and antibiotics.

In explorative analyses, cellular immunity against measles after early MMR vaccination and again after routine MMR vaccination at 15 months of age is evaluated as activated (interferon gamma producing) T cells recognizing selected measles-epitopes through ELIspot analyses [20]. Maternal antibodies, stress, and pollution have the potential to interfere with the immune response. Antibodies are assessed by blood samples from both infant and mother; pollution markers are tested in blood and urine, and acute and chronic stress markers are tested in blood, saliva, and hair. Furthermore, genetic association studies are planned based on buccal swaps to detect genes involved in the immune response to vaccination and stress response.

### Data collection

All data are entered directly into and stored in the e-crf and anonymized prior to publication of results.

#### Background information

A structured interview focusing on demographics, parental immunization against MMR, and risk factors such as atopic disposition and parental smoking habits as well as exposure to wild-type MMR is filled electronically by the parents prior to randomization.

#### Register-based data

Hospitalizations are identified using the Danish National Patient Registry. All Danish citizens have a unique social security number (CPR) that can be used to link data from the national Danish health registers at an individual level. The Danish Register of Medicinal Product Statistics contains CPR-number-based information about sale of all prescribed medicines in Denmark. The included children are followed in these registries until 24 months of age for breastfeeding, hospitalizations, and total medication use, including use of antibiotics, anti-asthmatics, and topical medication against eczema.

#### Clinical and paraclinical data

##### At inclusion (children participating without sample collection)

A thorough clinical examination of all children is performed before randomization and vaccination to document the health status of the child.

##### At inclusion (children participating with sample collection)

If the child is also participating in collection of biological samples (blood, urine, saliva, and hair), the samples are collected at the same contact prior to vaccination (300 MMR, 300 controls) but following a thorough child examination. See specifics in Table 1.

**Table 1:** Overview of handling, storage and primary use of the specimen collected

Sample	Handling	Storage	Measurement
Saliva	Collected in commercial kits 1) Saliva for DNA is not handled after collection. 2) Saliva for acute stress markers is centrifuged at 2000 RPM for 5 min at room temperature, pipetted to cryo tubes.	1) Room temperature 2) − 80 °C at the day of collection and stored until analysis	1) HLA tissue typing and genotyping by GWAS 2) Quantification of acute stress markers (alpha amylase, oxytocin and cortisol)
Hair	Hair is cut of as close to the scalp as possible and wrapped in tin foil and colored plastic bag. Preferable, 0.1–0.2 g of hair is sampled from the mothers and at least 0.025 g from infants.	Samples will be stored at room temperature in wrapping in a dry cabinet.	Mean cortisol concentration in last outgrown centimeter of hair.
Blood	Blood is collected in two different tubes. 1) Serum clot activator tubes for antibody measurement will be centrifuged at collection day and the serum will be pipetted to cryo tubes and frozen to − 20 °C. The following day the tubes will be moved to a − 80° freezer for storage. 2) Citrate tubes for T cell analysis will be transported at room temperature to collaborating laboratory for preparation for analysis for levels of activated T cells. If preparation cannot be performed on the day of collection the blood samples will be frozen and analysis will be performed within months of collection.	1) − 80 °C 2) − 196 °C	1) Antibody analyses will be performed in The Children’s Hospital Colorado. Antibody measurements will be performed in duplicates or triplicates if sufficient amount of 0.5 ml serum is present. IgM and IgG as well as PRNT will be performed in Colorado. 2) ELIspot analysis for activated T cells is performed and published by collaborating immunologists. 3) Quantification of pollution markers. Five PFASs (PFOS, PFOA, PFHxS, PFNA, PFDA) will be measured by solid-phase extraction and high-pressure liquid chromatography with tandem mass spectrometry.
Urine	Collected from mother in a cup and from child in a self-adhesive urine collection bag, pipetted into cryo tubes and frozen to − 80 °C at the day of collection and stored until analysis.	− 80 °C	Quantification of pollution markers.

##### Follow-up sample collection

One month after randomization at 7 months of age, and at 16 months of age 1 month after routine MMR vaccination at 15 months of age, blood samples are collected again in the children participating in the sample cohort for testing immunogenicity and cellular immune response (described in Table 1). If an MMR vaccine is administered before the age of 1 year, the parents are asked for permission to obtain an extra blood sample from the child 1 month after the administration. To ensure participation in follow-up visits, the families receive a reminder email the day before the appointment. Furthermore, the families will receive an email on the child’s 1-year birthday to remind them to contact their general practitioner regarding routine MMR vaccination and plan a visit 1 month later at the trial site.

##### Follow-up: adverse events

The families are recording adverse events on a diary including the most common adverse reactions related to MMR vaccination at 15 months, which they are introduced to by staff at the consultation. They are encouraged to report any experienced adverse event regardless of suspected relation to the vaccination. Six weeks after vaccination, all families are contacted by phone to obtain information from the diary.

A participant’s allocation code can be unblinded in case of suspected severe adverse events. Recording and reporting to the Danish Medicines Agency is performed according to rules and regulations [21]. Families are encouraged to contact the study staff spontaneously as early as possible if any suspected severe event occurs.

### Sample size consideration, power calculation

As there are two co-primary outcomes, there are also two power calculations. Sample size estimates are based on 95% confidence intervals and 80% power.

#### Immunogenicity

To detect a clinically significant difference of ≥ 0.3 SDS in the level of measles neutralizing antibodies between case children who receive MMR at 6 months of age and control children who receive placebo, a total of 500 samples per each of the three sample time points (at inclusion (500 children and 500 mothers), 1 month after experimental MMR vaccination at 6 months of age, and again 1 month after the MMR-booster vaccination scheduled at 15 months of age) are needed. To account for 20% dropout in follow-up blood samples 1 month after intervention and at 16 months of age, 600 mother-child pairs will be included in the collection of blood-samples.

#### Indirect effects of MMR

Ten percent of the child population in Denmark is expected to be hospitalized from 6 to 12 months of age [22]. To detect a 20% reduction in hospitalizations, 6426 children need to be included (3213 measles vaccinated children and 3213 control children).

For secondary and exploratory outcomes, all statistical tests will be adjusted for multiplicity.

### Monitoring and rules of termination

The Good Clinical Practice Unit (GCP) in the Capital Region monitors the trial independently of the sponsor. The trial was audited once in the beginning of the data collection period by the two GCP-units from western Denmark. The trial is monitored by an independent Data Safety Monitoring Board (DSMB) with two members who evaluate the study status and patterns of adverse events. The trial has no stopping rules as intervention is a widely used and safe vaccine recommended by authorities for use in infants as young as 6 months in case of measles epidemics and with no expected severe adverse events [16, 23], but the trial may be stopped by the DSMB after evaluation of the adverse event patterns after the inclusion of 2000, 4000, and 6000 participants, respectively.

### Statistical methods

Comprehensive statistical analysis plans are developed for the primary as well as for secondary outcomes; these plans are deposited with the DSMB before the allocation code is un-blinded and analyses are commenced.

#### Regarding first co-primary outcome: immunogenicity

A 5% significance level will be used. In each randomization group, the proportion of children with a non-protective level of antibodies according to standard cut-off values for each vaccine strain will be reported. In addition, the antibody geometric mean titer (GMT) in each randomization group will be reported. The primary estimate of the MMR effect is the geometric mean titer ratio (GMR) with 95% confidence interval obtained as the anti-logged coefficient from a linear regression with log-titer as outcome and randomization group, sex and gestational age as covariates. In case the antibody measurement assay is subject to upper or lower limits of detection, the GMR will be obtained by Tobit regression, which is a censored normal method. Non-detectable levels are not given a specific value but are instead regarded at censored with the true value being below/above the lower/upper limit. A prior study applied these methods to similar data on measles antibodies [24]. Level of maternal specific immunity (measles neutralizing antibodies by plaque-reduction neutralization test) at inclusion will be accounted for.

#### Regarding second co-primary outcome: indirect effects of vaccination

Cox proportional hazard models are used to estimate hazard ratios of hospitalizations due to infection after randomization/vaccination by allocation. The results are presented as hazard ratios (HR) with 95% confidence intervals (CIs) and *P* values of differences between groups. *P* values of < 0.05 are considered statistically significant. It is possible to follow-up 100% of the trial population in the public health registries. The children are censored at migration, 12 months of age, or death, whichever comes first. The analyses are stratified by sex and prematurity in accordance with the randomization procedure. In the main “intention-to-treat analysis” age at first hospitalization within the period from randomization to 12 months of age is analyzed according to randomization group. There will also be conducted a “per-protocol analysis” in which children who do not follow the allocation are excluded and time to first hospitalization is defined as time since vaccination for both the MMR-group and placebo group. No interim analyses are carried out, since data from the study is not linked to data from the health registries, and no samples analyzed until after LPLV.

## Discussion

As measles is a highly contagious and serious disease, means to improve protection are necessary. As passively acquired immunity through maternal antibodies ceases through the first 4 to 6 months of age, it renders the infant unprotected against measles, mumps, and rubella until the first MMR-vaccination, and thus, an optimization of first MMR vaccination time point is of great importance [5, 25]. If the timing of the MMR-vaccine is to be changed, it should be done only if there is the highest level of evidence that the MMR-vaccine elicits a sufficient immune response in the young infant resulting in improved immunity against MMR. Thus, sufficiently powered double-blind placebo-controlled randomized trials as the present are needed. To reduce the risk of for example healthy vaccinee bias and confounding by indication, which are inherently present in observational study designs [26], the randomized design is crucial in testing effects of vaccines and especially testing the hypothesis regarding potential indirect effects. High levels of maternal antibodies can compromise vaccine response, but other factors could affect the vaccine response in the young infant as well. The present trial aims to also qualify other factors leading to missing or insufficient vaccine response and provides insights to alternative responses than the humoral such as T cell activation.

## Trial status

The first day of inclusion was the 15th of April 2019. Last recruitment of participants is planned for April 2021. Latest approved protocol version is always accessible on the study homepage [27]. Current protocol version is version 7 dated 25th of March 2020.

## Steering group

The steering group consists of representatives from the two involved hospital’s children departments, a representative from Institute of Immunology and Microbiology, University of Copenhagen, and a representative from the Danish Center for Disease Control (SSI). The steering committee has the responsibility to monitor the trial and ensure progress of the trial according to the contract between Innovation Fund Denmark and the participating hospitals.

The DSMB consists of two experienced senior researchers. DSMB is responsible for monitoring trial safety.

## Supplementary information

**Additional file 1.** Questionnaire regarding backgroud information.

**Additional file 2.** Adverse events diary.

## Data Availability

The investigators on the trial will have access to the final trial dataset. In 2025, access to the data collection will be available for other researchers.

## Abbreviations

CBR: The Committees on Biomedical Research
DMA: Danish Medicines Agency
DSMB: Data safety monitoring board
eCRF: Electronic case report form
GMR: Geometric mean ratio
GMT: Geometric mean titer
LPLV: Last patient last visit
PRNT: Plaque reduction neutralization test
