# Supplementary material for: Measles-mumps-rubella vaccine at 6 months of age, immunology, and childhood morbidity in a high-income setting: study protocol for a randomized controlled trial
Source: Trials. 2020 Dec 10;21:1015. doi: 10.1186/s13063-020-04845-7 (PMC7727227; doi:10.1186/s13063-020-04845-7)
Supplement: Supplementary file 1 — Additional file 1. Questionnaire regarding backgroud information. [file 13063_2020_4845_MOESM1_ESM.docx]

**Questionnaire regarding backgroud information**

Date and time for filling out the questionnaire

Name

Social security number

Adress

Phone number

Cell phone number

Preferred contact hours

Email

In this research project we aim to investigate if infants will benefit from early MMR-vaccination already at 6 months of age. You can help us by letting your child participate and by answering some questions about your family.

(Unless anything else is stated the co-parent refers to the social/functioning father, in a homo-sexual relationship the co-parent).

Biological mothers’ age:_______________years Co-parents’ age:______________ years

Which (completed) education does the mother have?

-          Primary school ⁭

-          High school ⁭

-         Vocational education (e.g. craftsman/service jobs etc.) ⁭

-          Short higher education (e.g. computer scientist, construction technician, dental technician) ⁭

-          Medium lenght higher education (e.g. nurse/pedagouge/teacher/bachelor) ⁭

-          Long higher education (university) ⁭

-          Research education ⁭

-          Not applicable ⁭ (comment)______________________________________

Which (completed) education does the co-parent have?

-          Primary school ⁭

-          High school ⁭

-         Vocational education (e.g. craftsman/service jobs etc.) ⁭

-          Short higher education (e.g. computer scientist, construction technician, dental technician) ⁭

-          Medium lenght higher education (e.g. nurse/pedagouge/teacher/bachelor) ⁭

-          Long higher education (university) ⁭

-          Research education ⁭

-          Not applicable ⁭ (comment)______________________________________

Yearly income for the total house hold (DKK)_________________________________

<200000DKK 200000-400000DKK >400.000DKK

Is the mother or the biological co-parent of other ethnic origin than Danish? Yes No Do not know

Has the mother had measles Yes No Do not know

Is the mother vaccinated against measles Yes No Do not know

Has the mother had mumps Yes No Do not know

Is the mother vaccinated against mumps Yes No Do not know

Has the mother had rubella Yes No Do not know

Is the mother vaccinated against rubella Yes No Do not know

Has the co-parent had measles Yes No Do not know

Is the co-parent vaccinated against measles Yes No Do not know

Has the co-parent had mumps Yes No Do not know

Is the co-parent vaccinated against mumps Yes No Do not know

Has the co-parent had rubella Yes No Do not know

Is the co-parent vaccinated against rubella Yes No Do not know

*Now, a few questions about smoking habits in the family*

Does the mother smoke (inside and/or outdoors) Yes, daily Yes, weekly Yes, but rarer than once a week No Do not know

Did the mother smoke while she was pregnant with this child? (Also include the very early pregnancy)

Yes No Do not know

Does the co-parent smoke (inside and/or outdoors) Yes, daily Yes, weekly Yes, but rarer than once a week No Do not know

Did the co-parent smoke while she was pregnant with this child? (Also include the very early pregnancy)

Yes No Do not know

# Now, some questions regarding pets.

Have you had pets during pregnancy?

Yes No Do not know

*If yes: What kind: Animals with fur*⁭ Exclusively animals without fur ⁭ Do not know ⁭

*A few questions about your home:*

Do mother and co-parent live together (Population register address)?

Yes No Do not know

Do you live in (population register address):

Owner occupied housing⁭ Housing cooperative ⁭ Rental ⁭

*Here are some questions about respiratory diseases and allergies in your family:*

*(Only DISEASES diagnosed by a doctor should be registered. It should also be registered if the person has grown from his symptoms. Parents is mean BIOLOGICAL parents, full siblings and half siblings to the participating child).*

| Sibling number and parent title | 1.sibling (oldest) | 2. sibling | 3. sibling | 4. sibling | 5. sibling | 6. sibling | Mor  (biologisk) | Far  (biologisk) |
| --- | --- | --- | --- | --- | --- | --- | --- | --- |
| Birth year (year) |  |  |  |  |  |  |  |  |
| Food allergy (X) |  |  |  |  |  |  |  |  |
| Atopic dermatitis (X) |  |  |  |  |  |  |  |  |
| Hay fever (X) |  |  |  |  |  |  |  |  |
| Asthma (X) |  |  |  |  |  |  |  |  |
| Asthmatisk bronchitis (X) |  |  |  |  |  |  |  |  |
| Followed the recommended vaccination program? |  |  |  |  |  |  | Not relevant | Not relevant |
| Adverse reactions following vaccines: 1= None, 2 = Insignificant, 3 = Serious |  |  |  |  |  |  | Not relevant | Not relevant |

*Finally, there are some questions that can help measure stress levels:*

**WHO Five Well-being Index (1999 version, © Psychiatric Research Unit, WHO Collaborating Center for Mental Health, Frederiksborg General Hospital, DK-3400 Hillerød)**

*Please tick each of the 5 statements; A cross in the field that comes closest to how you have felt in the past two weeks. A higher number means greater well-being.*

*Example: If you have felt happy and in a good mood for a little more than half the time in the last two weeks, put the cross in the box with the number 3 in the upper right corner.*

| In the last two weeks… | All the time | Most of the time | A little bit more than half the time | A little bit less than half the time | A bit of the time | At no time point |
| --- | --- | --- | --- | --- | --- | --- |
| Have I been happy and in a good mood | *5* | *4* | *3* | *2* | *1* | *0* |
| have I felt calm and relaxed | *5* | *4* | *3* | *2* | *1* | *0* |
| have I felt active and energic | *5* | *4* | *3* | *2* | *1* | *0* |
| have I woken up fresh and rested | *5* | *4* | *3* | *2* | *1* | *0* |
| have my weekdays been occupied with stuff that interests me | *5* | *4* | *3* | *2* | *1* | *0* |

*Score calculation*

*To calculate score numbers, add the numbers in the checked boxes together and multiply by 4. You get a number between 0 and 100. The higher score the greater the well-being. If the number is 50 or less, you are at risk for stress or depression, and we would recommend contacting your own doctor about this.*

*Now, there are no more questions. Do you have any comments on the questions? (Write down if necessary)*

Comments:

Filled out by_____________________________________________________________________

Thank you so much for the help!
