# Supplementary material for: Measles-mumps-rubella vaccine at 6 months of age, immunology, and childhood morbidity in a high-income setting: study protocol for a randomized controlled trial
Source: Trials. 2020 Dec 10;21:1015. doi: 10.1186/s13063-020-04845-7 (PMC7727227; doi:10.1186/s13063-020-04845-7)
Supplement: Supplementary file 2 — Additional file 2. Adverse events diary. [file 13063_2020_4845_MOESM2_ESM.pdf]

Bivirkningsdagbog ved studiet: Er det bedre at vaccinere børn mod mæslinger, fåresyge og røde hunde allerede ved 6-måneders alderen?

Bivirkningsdagbog for ___________________________

Eventuelle bivirkninger registreres i 6 uger efter injektion (anfør datoer og sæt kryds i symptom), hvorefter vores personale kontakter jer telefonisk med spørgsmål. Hvis jeres barn **indlægges** med noget, I mistænker, kan være bivirkninger, bedes I kontakte os på tlf.: 35 45 25 03.

**Dato for bivirkning**														
**Forkølelse**														
**Løbende næse**														
**Diare og opkastning**														
**Udslæt**														
**Feber (38,5^{o} eller højere) Anfør højest målte temp.**														
**Rødme, ømhed og hævelse på indstiksstedet**														
**Blåt mærke på indstikssted**														
**Kløe på indstiksstedet**														
**Feberkramper**														
**Fald i antal blodplader (målt hos læge)**														
**Har I haft kontakt til læge gr. bekymring om bivirkninger?**														
**Andet**														

Vi vil forsøge at træffe jer telefonisk i uge ______________________
